# Influence of Total Running Experience on Lower Leg Variability: Implications for Control and Performance in Male Athletes

**DOI:** 10.3390/sports13020058

**Published:** 2025-02-17

**Authors:** Jared Steele, Iain Hunter

**Affiliations:** 1 Department of Human Evolutionary Biology, Harvard University, Cambridge, MA 02138, USA; 2 Department of Exercise Sciences, Brigham Young University, Provo, UT 84602, USA; iain_hunter@byu.edu

**Keywords:** kinematic variability, running experience, performance

## Abstract

This study investigates the relationship between total running experience, defined as cumulative years of running multiplied by weekly mileage, and variability in lower leg joint kinematics during treadmill running. Twenty-seven male athletes participated, running while kinematic and kinetic data were collected. Linear regression revealed significant negative correlations between total running experience and variability in both knee and ankle joint range of motion (ROM). Specifically, ankle ROM variability (*p* = 0.001, R^2^ = 0.35) and knee ROM variability (*p* = 0.002, R^2^ = 0.32) were reduced in runners with more experience. A stepwise regression model further identified ankle ROM variability as a significant predictor (*p* = 0.033), explaining 44.25% of the variance in total running experience. A significant positive correlation between running experience and instantaneous vertical loading rate (IVLR) (*p* = 0.025, R^2^ = 0.15) suggests that more experienced runners generate higher load rates. These findings indicate that more experienced runners exhibit more consistent and stable movement patterns, reflecting refined motor control. The results support the hypothesis that greater running experience is associated with reduced variability in movement patterns within a controlled environment, providing insights into the mechanisms that could contribute to enhanced performance and injury prevention.

## 1. Introduction

Running is widely recognized for its health benefits, yet it carries notable risks, particularly for novice runners. Overuse injuries remain a prevalent concern, with reported incidence rates ranging from 19% to 79%, often attributed to factors such as weekly mileage and cumulative years of running [1,2,3]. Contrary to traditional beliefs, recent research suggests that running experience may not be a factor in injury risk for novice runners [4]. This finding has redirected attention toward alternative explanations for the observed variability in injury rates across experience levels.

Advances in biomechanics highlight the role of motor control strategies, which adapt and evolve with experience [5]. These adaptations influence coordination variability, defined as the movement pattern differences between segments, which is gaining recognition as a critical aspect of running biomechanics [6,7]. While this study quantifies variability using the standard deviation of sagittal plane joint kinematics, alternative mathematical approaches exist to assess movement variability. Lyapunov exponents measure the divergence of small perturbations in movement trajectories, providing insights into stability and adaptability [8]. Approximate entropy and sample entropy quantify movement complexity and predictability, while fractal analysis methods such as Detrended Fluctuation Analysis (DFA) assess long-range correlations in movement patterns. Additionally, vector coding techniques have been widely used to evaluate segmental coordination variability, particularly in running biomechanics [9,10]. These approaches offer deeper insights into inter-segmental interactions, which could be valuable for future investigations into motor control adaptations in experienced runners. Although these approaches provide advanced perspectives on variability, standard deviation remains a useful metric for comparing variability across different experience levels in running biomechanics. Empirical evidence suggests that trained runners demonstrate reduced movement variability compared to novices, indicating that cumulative weekly mileage and years of running, collectively termed ‘total running’, may refine motor patterns and stabilize coordination [8].

Movement variability can be categorized into two distinct forms: (1) skill acquisition variability, where higher variability reflects inconsistent motor control during the learning process, and (2) functional variability, where controlled movement adjustments enable experienced runners to optimize performance and adaptability to environmental changes. This distinction has been studied in motor control research [11,12], with findings suggesting that reduced variability in experienced runners reflects enhanced coordination and efficiency, rather than motor inflexibility [12]. These forms of variability play a role in running biomechanics, where the transition from high to controlled variability marks the progression from learning to skilled running performance. This dual function of variability, as a marker of motor refinement and adaptability, is consistent with findings across other sports, emphasizing the necessity for focused investigations in running biomechanics [13].

While movement variability has been extensively studied in sports such as cycling, race walking, and swimming [14,15,16], findings in running remain inconsistent, revealing a gap in understanding how experience shapes running biomechanics. Bridging this gap is essential to resolving the mechanisms responsible for possible running-related injuries and performance [17,18]. In addition to sagittal joint angles at the knee and angle, ankle angle at initial contact and instantaneous vertical loading rate (IVLR) are also markers of skill adaptation and stability in running. The ankle angle at initial contact is critical for assessing shock attenuation during the stance phase, with novice runners often exhibiting less stable landing mechanics [19]. IVLR, a measure of force application during early stance, has been correlated with training exposure and neuromuscular control, offering insights into injury susceptibility and motor refinement [20]. Although variability has been shown to decrease with experience, it is unlikely that this reduction continues indefinitely in a linear fashion. Research on motor learning suggests that movement variability may reach a plateau beyond which additional experience does not significantly alter coordination patterns [21]. This concept is well documented in skill acquisition studies, where performance improvements stabilize over time as an optimal movement strategy emerges. Future studies should investigate whether running experience follows a similar asymptotic trajectory, in which variability reductions become negligible beyond a certain threshold of training exposure.

This current study examines the association between total running experience on lower extremity variability during controlled treadmill running. This study also includes exploratory metrics, such as $\overset{.}{V}O_{2}$ and IVLR, to examine whether physiological and mechanical factors also influence kinematic variability in runners [19,20]. We hypothesize that greater running experience will be associated with reduced knee and ankle variability, reflecting a more stable and refined motor control strategy. Given that the knee and ankle play primary roles in impact absorption and propulsion, they are more sensitive to training effects than the hip [9,22]. While the hip contributes to global movement control, prior research suggests that distal joint variability is more directly influenced by long-term running exposure, making the knee and ankle more appropriate targets for analysis.

Research has shown that segmental coordination patterns evolve with experience, particularly at the shank and foot, where greater adaptability emerges over time [9]. However, at the joint level, running experience is typically associated with reduced movement variability, as motor refinement minimizes unnecessary motion [22]. By focusing on joint kinematics, rather than inter-segmental coordination, this study aims to evaluate how running experience is associated with motor control strategies at individual joints. Further, the inclusion of ankle angle at initial contact as a stance-phase-specific metric aligns with its role in assessing sagittal plane stability and control during running.

As a secondary focus, this study investigates metabolic factors, specifically sub-maximal $\overset{.}{V}O_{2}$, to explore their potential relationship with movement variability in runners. Sub-maximal $\overset{.}{V}O_{2}$ measured during steady-state running, serves as a proxy for running economy and reflects the efficiency of neuromuscular and metabolic systems under controlled conditions. Previous research has shown that improved running economy is associated with refined motor patterns and enhanced performance, suggesting that sub-maximal $\overset{.}{V}O_{2}$ may play a role in reducing variability during repetitive tasks [23,24].

## 2. Materials and Methods

A total of 27 male athletes were recruited for this study. All participants had prior experience running on the laboratory treadmill and completed a questionnaire documenting recent injuries, weekly mileage, and cumulative years of running. Inclusion criteria required participants to report no history of chronic overuse injuries within the six months preceding the study. Participant demographics and training characteristics are summarized in Table 1. Written informed consent was obtained from all participants before inclusion, and the study protocol was approved by the Brigham Young University Institutional Review Board (IRB #IRB2021-302).

**Figure 1:**
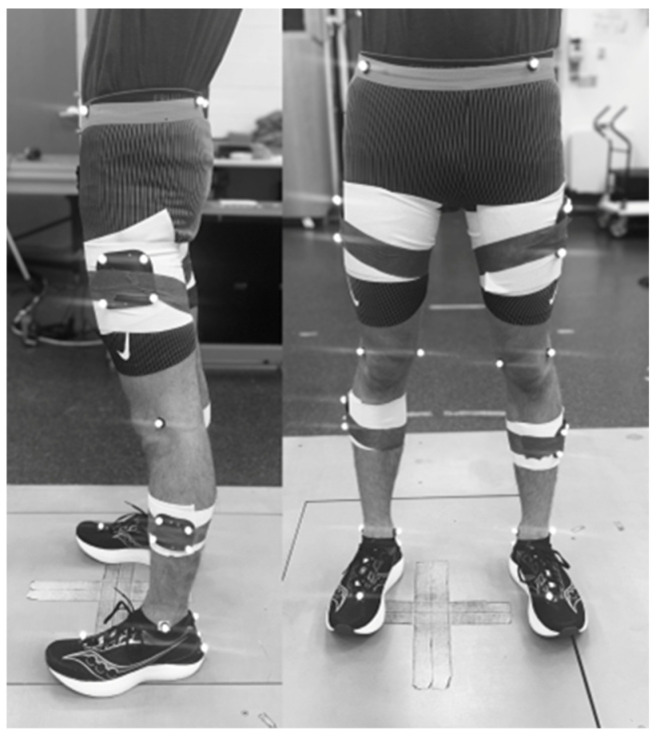
Marker setup and placement for all subjects.

Three-dimensional kinematic data were collected using a 13-camera Vicon motion capture system (Vicon Motion Systems, Oxford, UK) sampling at 240 Hz, while kinetic data were recorded from a force-instrumented treadmill (Bertec, Columbus, OH, USA) sampling at 960 Hz. Prior to each participant’s data collection, the motion capture system was calibrated following the manufacturer’s guidelines to ensure optimal accuracy of marker tracking. Retro-reflective markers (n = 24) were placed on anatomical landmarks of the lower body, including the medial and lateral epicondyles, medial and lateral malleoli, the first metatarsal head, and a triad on the heel (Figure 1). To ensure consistency in marker placement, the same researcher applied the markers for all participants. Marker placement was verified by a second researcher to reduce potential error. All participants were distance runners with low body fat percentages, which facilitated the accurate identification of bony landmarks. Rigid marker clusters with four non-collinear reflective markers were secured to the lateral aspects of the thigh and shank segments for tracking. Pelvic kinematics were captured using markers positioned at the bilateral anterior superior iliac spines (ASISs) and posterior superior iliac spines (PSISs). To assess metabolic responses, participants wore a portable metabolic measurement system (Cosmed K5, Rome, Italy) following marker placement.

Participants completed a 5 min warm-up at a constant speed of 3.83 m/s to acclimate to the setup and achieve steady-state $\overset{.}{V}O_{2}$ levels. Following the warm-up, participants performed a 10 min treadmill running trial at the same speed. Kinematic and kinetic data were captured during the final 30 s of minutes 3, 5, 7, and 9, resulting in approximately 30 strides per collection interval. Metabolic data were continuously recorded throughout the trial and averaged over consecutive 10 s periods. Steady-state $\overset{.}{V}O_{2}$ values were confirmed, with the final 3 min average used to represent the entire trial. Analyses focused on data collected after the initial 3 min to account for the stabilization of running biomechanics.

Joint kinematic data were derived from three-dimensional marker positions captured during running trials. Marker positions were processed using a 20 Hz low-pass, second-order Butterworth filter in Visual3D (C-Motion, Germantown, MD, USA). Ankle angle at initial contact was calculated as the sagittal plane ankle joint angle at the moment of initial contact, defined as the first frame when vertical ground reaction force exceeded 30 N. This approach was automated within Visual3D software v2024.06.1, ensuring consistent identification across trials. Instantaneous vertical loading rate (IVLR) was calculated from the vertical ground reaction force (GRF) data to capture impact mechanics during the stance phase. IVLR was defined as the derivative of the vertical GRF just prior to the impact peak (IP). For participants without an identifiable IP—commonly observed in forefoot strike (FFS) patterns—IVLR was taken at 13% of stance [25].

### Statistical Analysis

All initial data visualizations and statistical analyses were performed using MATLAB (Version R2023b, 23.2, The MathWorks Inc., Natick, MA, USA) and R (Version 2024.04.2+764 (2024.04.2+764), R Core Team, 2024). Segment variability was quantified as the standard deviation of joint kinematic data over each 30 s collection period. Descriptive statistics are reported as means and standard deviations, and data normality was verified using the Shapiro–Wilk test. Relationships between total running experience and joint kinematic variability were assessed using linear regression analyses. To address potential false discovery rates, the Benjamani–Hochberg method [26] was applied to regression-derived *p*-values. This method controls the false discovery rate, which is the expected proportion of incorrect rejections among all rejected hypotheses, creating a balance between statistical power and error rate. Significant predictors were incorporated into the stepwise regression model, using the Akaike Information Criterion (AIC) to identify the final model [27]. The AIC is a model selection criterion that evaluates the relative quality of statistical models for a given dataset by balancing model fit with complexity, which helps avoid overfitting. Statistical significance was inferred from *p* < 0.05.

## 3. Results

### 3.1. Correlation Analysis

Prior to regression analyses, Pearson correlation coefficients were calculated to assess the relationships between total running experience and key variables. Significant negative correlations were observed between total running experience and knee ROM variability (r = −0.57, *p* = 0.002), ankle ROM variability (r = −0.59, *p* = 0.001), and ankle angle at initial contact variability (r = −0.49, *p* = 0.008). A significant positive correlation was found between total running experience and IVLR (r = 0.39, *p* = 0.025).

### 3.2. Linear Regression Analysis

Before conducting regression analyses, we assessed the normality of key dependent variables using the Shapiro–Wilk test to confirm the appropriateness of parametric statistical methods. The results indicated that knee ROM variability (W = 0.973, *p* = 0.696), ankle ROM variability (W = 0.953, *p* = 0.255), ankle angle at initial contact variability (W = 0.969, *p* = 0.712), and instantaneous vertical loading rate (IVLR) (W = 0.983, *p* = 0.925) all met the assumption of normality (*p* > 0.05). Given this, parametric linear regression analyses were deemed appropriate for further statistical testing.

**Figure 2:**
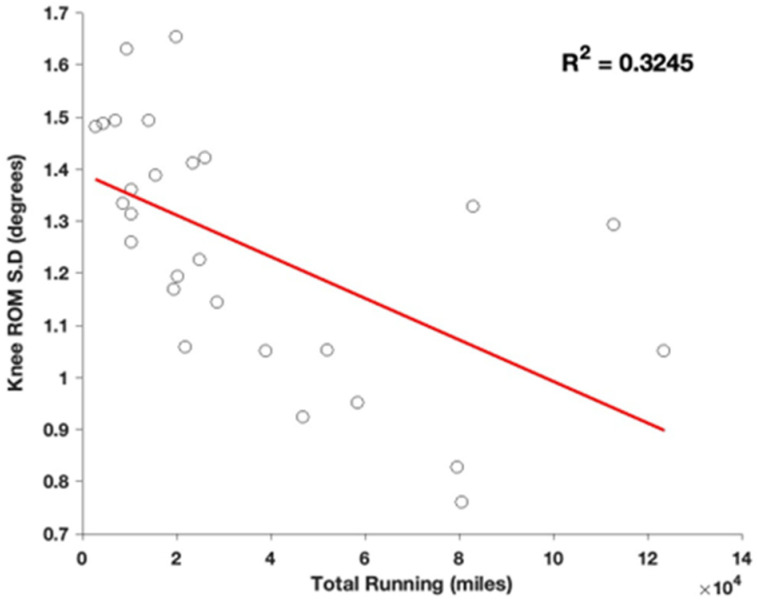
Regression comparing Knee ROM variability, defined as standard deviation across the collection, compared to total years spent running.

**Figure 3:**
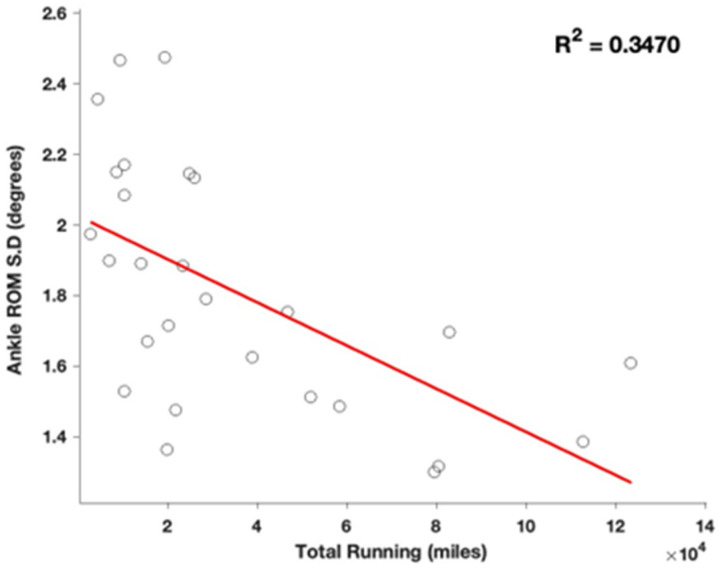
Regression comparing ankle ROM variability, defined as standard deviation across the collection, compared to total years spent running.

Initial linear regression analyses indicated significant negative correlations between total running experience and variability measures in the lower extremity joints. Specifically, significant relationships were found for knee range of motion (ROM) variability (β = −0.57, *p* = 0.002, R^2^ = 0.32) (Figure 2), ankle ROM variability (β = −0.59, *p* = 0.001, R^2^ = 0.35) (Figure 3), and ankle angle at initial contact variability (β = −0.49, *p* = 0.008, R^2^ = 0.24). Additionally, there was also a significant positive correlation between total running and instantaneous vertical loading rate (IVLR) (β = 0.39, R^2^ = 0.15; *p* = 0.025), suggesting that increases in running experience lead to greater load rates.

### 3.3. Stepwise Regression Analysis

A stepwise regression analysis was performed. The initial model included knee ROM variability, ankle angle at initial contact, and ankle ROM variability. During the stepwise process, ankle angle at initial contact was removed due to its higher *p*-value, resulting in a final model that included knee ROM variability and ankle ROM variability as predictors.

In the final model, ankle ROM variability remained a significant predictor (*p* = 0.033), indicating that greater total running experience is associated with reduced ankle range of motion variability. Although knee ROM variability was not statistically significant at the conventional 0.05 level (*p* = 0.053), it approached significance, suggesting a trend where increased running experience may be linked to reduced knee range of motion variability. The overall model explained 44.25% of the variance in total running experience (adjusted R^2^ = 0.396, *p* < 0.001).

## 4. Discussion

This study investigated the association between total running experience, cumulative years of running and weekly mileage, and lower leg joint kinematic variability during controlled treadmill running, a setup that contrasts with prior research conducted in outdoor or non-standardized environments [6,9,28]. We hypothesized that increased total running experience would be associated with reduced variability in joint movements, reflecting a more refined motor control strategy within a controlled environment such as treadmill running. Our findings support this hypothesis, revealing significant negative correlations between total running experience and lower leg joint variability, consistent with Hafer et al. [9], who observed reduced variability in experienced runners, and Nakayama et al. [28], who identified variability differences between trained and untrained individuals.

However, the significant positive correlation between IVLR and running experience warrants further consideration. While greater load rates might indicate enhanced mechanical efficiency or ability to apply greater forces, they may also reflect an increased risk of injury, contradicting the injury-prevention narrative typically associated with reduced variability. This emphasizes the complexity of motor control adaptations and their implications for long-term injury risk.

### 4.1. Knee and Ankle Variability

The significant negative association between total running experience and ankle ROM variability aligns with findings by Nakayama et al. [28] and Hafer et al. [9], who reported greater stability and reduced variability in distal joints, likely reflecting refined motor control strategies. This finding supports our rationale for including ankle angle at initial contact, a metric that reflects sagittal plane kinematics, as it aligns with prior studies emphasizing the role of distal joint coordination in improving motor control strategies with experience. This finding aligns with previous research indicating that experienced runners develop more stable and efficient motor patterns because of prolonged training and exposure to repetitive tasks [11,29]. Similar trends were observed in the knee joint, where the relationship between total running experience and knee ROM variability approached significance. This suggests that increased running experience may contribute to reduced knee variability, potentially reflecting a more consistent gait pattern. However, the marginal significance of this result highlights the need for further investigation. The greater reductions observed in ankle compared to knee variability may reflect a differential influence of training on distal versus proximal joints, supporting theories that motor refinement may primarily affect structures farther from the center of mass. Such findings are in line with earlier studies, which have reported that experienced athletes in other sports exhibit reduced movement variability as they refine their motor control strategies through practice [3]. It is interesting that variability is reduced by experience more at the distal joints (ankle) than the proximal joints (knee and hip). This suggests that experience and motor learning may have less of an impact when closer to one’s center of mass.

**Figure 4:**
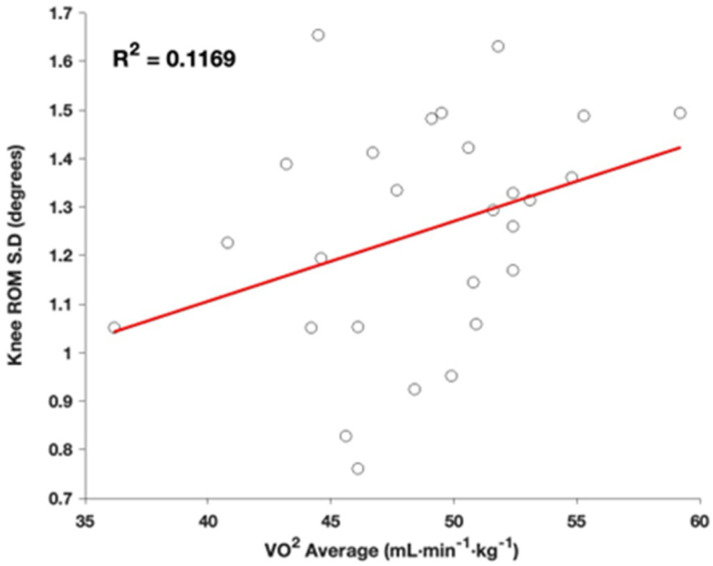
Regression of knee ROM variability, defined as standard deviation across the collection, compared to oxygen consumption of the individuals.

Although our primary focus was on lower limb variability, our analysis revealed no relationship between $\overset{.}{V}O_{2}$ and variability (Figure 4). This lack of association between $\overset{.}{V}O_{2}$ and variability may suggest that motor learning is more influenced by cumulative task repetition (as captured by total running experience) than by metabolic efficiency or performance capabilities. Variability metrics and oxygen consumption values can be seen in Table 2.

### 4.2. The Role of Motor Control and Adaptability

Our findings contribute to the ongoing debate on variability as a marker of motor control, supporting Latash et al. [11], who described variability as balancing refinement and adaptability. The inclusion of instantaneous vertical loading rate (IVLR) as a metric further contextualizes this balance, reflecting the biomechanical response to impact forces during early stance. This metric aligns with established protocols for quantifying impact loading and variability, particularly for mid-stance transitions [25]. However, the controlled treadmill environment in this study limits extrapolation to dynamic or unpredictable running conditions. While some researchers argue that reduced variability indicates a more refined and efficient motor pattern [30], others suggest that a certain level of variability is necessary for adaptability to changing conditions [31]. The trend towards reduced variability with increased experience in our study suggests that experienced runners may achieve a balance between stability and adaptability, optimizing their movement patterns for both efficiency and responsiveness to environmental perturbations. This dual role of variability, both as a marker of motor refinement and as a potential indicator of adaptability, reinforce the findings from previous studies on movement variability in other athletic contexts, such as race walking and swimming [15,16]. While there were no environmental perturbations in this study as the subject ran at the same pace on a treadmill, it is unclear how years of training would contribute to changes in variability if we were to increase either speed or grade of running.

### 4.3. Comparison with Other Sports

Our findings align with studies in other cyclical sports, such as cycling and swimming, where experienced athletes demonstrate reduced movement variability [14,16]. However, running’s unique biomechanical demands, including load impact and dynamic joint behavior, differentiate it from these sports [15]. The observed differences between ankle and knee variability reductions further highlight the unique motor control adaptations required in running, as opposed to sports like swimming, where dynamic loading forces are less critical. This consistency across different sports underscores the importance of practice and experience in refining motor control strategies, leading to more stable and efficient performance. The similarity in these findings across various forms of locomotion suggests that the principles governing the relationship between experience and variability may be generalizable across different types of physical activities.

### 4.4. Limitations and Future Research

Despite the strengths of this study, there are limitations that need addressing. The sample consisted solely of male runners, potentially limiting generalizability to female runners or older individuals, who are known to exhibit distinct variability patterns [10]. Future research should expand to more diverse populations and assess variability in outdoor or non-standardized running conditions, where motor control demands may differ significantly. Prior research has suggested that gender and age can influence running mechanics and coordination variability [10]. Future research should explore these relationships in more diverse populations and possibly how different training regimens impact variability in joint kinematics. Our reliance on metrics such as ankle angle at initial contact, IVLR, and ROM variability, though justified by prior research, shows the need for longitudinal studies to validate their predictive utility for motor control and injury prevention in running. Our study does not assess whether reductions in kinematic variability continue indefinitely or whether they plateau after reaching an optimal level of motor control. Research in motor learning suggests that skilled movement patterns typically stabilize over time, resulting in diminishing returns in variability reduction as experience increases [21]. Longitudinal studies or statistical modeling approaches could help determine whether highly experienced runners exhibit an asymptotic reduction in variability, where further training no longer leads to meaningful biomechanical adaptations. Future research could also explore the combined effects of speed, fatigue, and terrain variability, which may reveal more nuanced relationships between training experience and motor control.

## 5. Conclusions

Our study supports the hypothesis that increased total running experience is associated with reduced variability in lower leg joint movements during running within a controlled environment. These findings suggest that experienced runners develop more consistent and stable movement patterns, which may contribute to improved performance and reduced injury risk. This study demonstrates that total running experience is associated with reduced lower leg variability, reflecting refined motor control strategies. These findings contribute to a broader understanding of variability as both a marker of stability and adaptability in cyclical sports. While experienced runners exhibit more consistent movement patterns, future research should explore how variability adapts to external perturbations, such as uneven terrain or fatigue. By advancing our knowledge of motor control in running, this research offers valuable insights for optimizing training strategies and reducing injury risks in athletes.

## Figures and Tables

**Table 1:** Subject characteristics.

Subject	Age (Years)	Height (cm)	Mass (kg)	Weekly Mileage (Miles)	Years Running (Years)	Total Running (Miles)	$\overset{.}{V}O_{2}$ (mL·min^−1^·kg^−1^)
1	49	177.8	68.2	70	31	2170	51.6
2	28	177.8	67.3	25	15	375	52.4
3	20	185.42	71.4	55	1	55	49.1
4	32	180.34	68.2	90	17	1530	45.6
5	23	188	79.5	50	10	500	50.6
6	21	170.18	65.9	48	8	384	44.5
7	24	177.8	64.1	15	11	165	47.7
8	30	177.8	71.8	60	15	900	48.4
9	25	167.6	63.6	30	9	270	59.2
10	18	180.3	65.9	45	4	180	51.8
11	18	177.8	55.5	50	4	200	53.1
12	20	180.3	68.2	60	7	420	50.9
13	26	188	70.3	20	15	300	43.2
14	18	175.3	57.2	50	4	200	54.8
15	37	180.3	80.3	40	25	1000	46.1
16	32	177.8	79.5	10	20	200	52.4
17	30	182.8	68.0	30	15	450	46.7
18	42	175.26	63.1	95	25	2375	36.2
19	25	182.88	82.0	65	6	390	44.6
20	21	181	74.0	75	10	750	44.2
21	21	172.5	63.0	60	8	480	40.8
22	19	178	66.3	45	3	135	49.5
23	44	182	69.4	50	31	1550	46.1
24	21	179.5	58.6	14	6	84	55.3
25	42	183.5	88.5	55	29	1595	52.4
26	23	180.5	73.5	55	10	550	50.8
27	45	177.5	65.8	45	25	1125	49.9
	27.92 (9.38)	179.18 (4.65)	69.22 (7.73)	48.41 (21.31)	13.48 (9.05)	708.32 (651.2)	48.81 (4.87)

**Table 2:** Biomechanical variables, oxygen consumption, and individual total mileage of each subject.

Subject	Total Running (Miles)	$\overset{.}{V}O_{2}$ (mL·min^−1^·kg^−1^)	IVLR	Knee Range of Motion S.D (Degrees)	Ankle Range of Motion S.D (Degrees)
1	112,840	51.6	146.7	1.29	0.62
2	19,500	52.4	96.1	1.17	0.61
3	2860	49.1	69.6	1.48	0.88
4	79,560	45.6	87.6	0.83	0.84
5	26,000	50.6	114.4	1.42	1.04
6	19,968	44.5	123.9	1.65	0.66
7	8580	47.7	101.6	1.34	0.77
8	46,800	48.4	144.6	0.92	0.57
9	14,040	59.2	58.9	1.49	0.84
10	9360	51.8	68.6	1.63	0.97
11	10,400	53.1	77.4	1.31	0.63
12	21,840	50.9	112.1	1.06	0.71
13	15,600	43.2	118.4	1.39	0.72
14	10,400	54.8	96.0	1.36	0.97
15	52,000	46.1	121.1	1.05	0.62
16	10,400	52.4	125.1	1.26	0.72
17	23,400	46.7	86.8	1.41	0.77
18	123,500	36.2	117.4	1.05	0.59
19	20,280	44.6	57.1	1.19	0.88
20	39,000	44.2	113.4	1.05	0.58
21	24,960	40.8	94.9	1.23	0.48
22	7020	49.5	48.1	1.49	0.85
23	80,600	46.1	85.0	0.76	0.54
24	4368	55.3	85.0	1.49	0.84
25	82,940	52.4	100.7	1.33	0.86
26	28,600	50.8	124.7	1.14	0.89
27	58,500	49.9	165.6	0.95	0.49
	35,308 (33,514)	48.81 (4.87)	101.51 (28.64)	1.25 (0.23)	0.74 (0.16)

## Data Availability

The raw data supporting the conclusions of this article will be made available by the authors on request.
